# Optical Film with Microstructures to Regulate Viewing Angle of HUDs

**DOI:** 10.3390/mi16060714

**Published:** 2025-06-16

**Authors:** Qibin Feng, Xiangjun Li, Chunhui Chen, Guoqiang Lv, Zi Wang

**Affiliations:** 1 Special Display and Imaging Technology, Innovation Center of Anhui Province, Anhui Province Key Laboratory of Measuring Theory and Precision Instrument, Hefei University of Technology, Hefei 230009, China; fengqibin@hfut.edu.cn; 2 School of Instrument Science and Opto-electronics Engineering, Hefei University of Technology, Hefei 230009, China; 18298025766@163.com (X.L.); 15570123076@163.com (C.C.); guoqianglv@hfut.edu.cn (G.L.)

**Keywords:** head-up display, viewing angle, microstructure, optical film

## Abstract

Head-up displays (HUDs) can effectively enhance driving safety by projecting information—such as speed and maps—onto the windshield, thereby reducing blind spots caused by drivers looking down. As drivers need to observe road conditions within a wider horizontal viewing field, and considering that the observed angle in a vertical direction is relatively small, it becomes reasonable for an HUD to present a larger horizontal viewing angle than vertical viewing angle. This paper proposes a method to independently regulate the horizontal and vertical viewing angles. The original microstructure morphology is modeled as an ellipsoid, and the curvatures of the ellipsoid’s major and minor axes are calculated according to the required viewing angles. The simulation results show that the horizontal viewing angle corresponding to 85% of the maximum luminance increases from 2° without the film to 20° with the film, while the vertical viewing angle increases from 2° to 8°. The optical film with the designed microstructures is prepared and measured. The practical measurement results indicate that the tested horizontal and vertical viewing angles exhibit significant differentiation. At 85% of the maximum luminance, the horizontal viewing angle increases from 2° without the film to 23° with the film, while the vertical viewing angle increases from 2° to 10°. These results meet the requirements for independently regulating horizontal and vertical viewing angles and widening the horizontal viewing angle.

## 1. Introduction

A head-up display (HUD) can project driving information—such as speed and maps—onto the windshield to reduce blind spots caused by drivers looking down [1,2,3]. In practical driving scenarios, as drivers need to observe road conditions within a wider horizontal viewing field, and considering that the observed angle in a vertical direction is relatively small, it becomes reasonable for an HUD to present a larger horizontal viewing angle than vertical viewing angle [4,5]. However, the horizontal and vertical viewing angle curves of current HUDs are typically similar. It is therefore necessary to have a solution that independently regulates the horizontal and vertical viewing angles to widen the horizontal viewing angle.

Optical film with microstructures is one solution to regulate viewing angles. Feng et al. designed a microstructure profile to deflect the maximum luminance of a liquid crystal display in a normal direction to certain angles [6,7]. Tuo et al. utilized microstructures to enhance the display luminance of an HUD [8]. Zhang et al. combined free-form surfaces and microstructures to optimize the display performance of an HUD [9]. However, all the above-mentioned studies could not regulate the vertical and horizontal viewing angles separately, making it completely impossible to extend the horizontal viewing angle while keeping the vertical viewing field unchanged.

This paper proposes a design method for microstructures on optical film to independently regulate horizontal and vertical viewing angles. The optical film was prepared to verify the proposed design.

## 2. Theoretical Design of Microstructures

### 2.1. HUD Optical System

**Figure 1:**
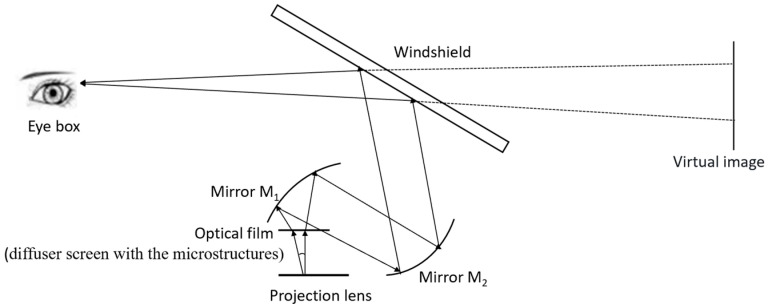
HUD optical system with optical film.

An HUD optical system based on a digital micro-mirror device (DMD) usually includes a projection lens, two mirrors, a diffuser screen, and a windshield [10,11], as shown in Figure 1. The mirrors are free-form surfaces designed to match the windshield curvature, folding the optical path and reducing the HUD volume. The image emitted by the projection lens is first formed on a diffusion screen. The formed image is then reflected by the mirrors and the windshield to reach the viewer’s eyes, creating a virtual image in front of the vehicle.

As described in Section 1, the microstructures help widen the horizontal viewing angle. This paper first designs the profile of the microstructure; the microstructure array is then fabricated on a diffuser screen, hereafter referred to as the optical film.

### 2.2. Microstructure Profile Design

**Figure 2:**
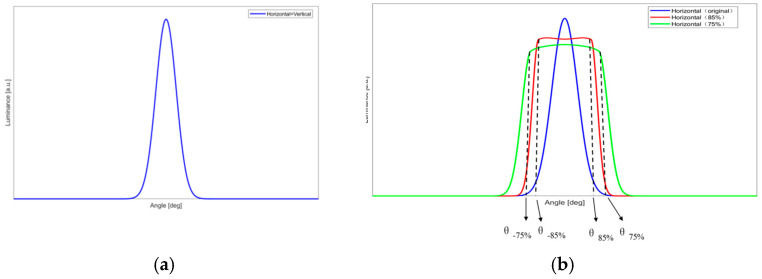
Viewing angle curves presented by (**a**) common HUD; (**b**) HUD with optical film.

Figure 2 shows the common (Figure 2a) and expected (Figure 2b) viewing angle curves. In Figure 2a, the horizontal angle curve is identical to the vertical angle curve. As shown in Figure 2b, the horizontal viewing angle becomes significantly wider.

In this paper, the required horizontal and vertical viewing angles serve as the design specifications. However, their influence on luminance must also be considered. It is reasonable that as the viewing angle increases, the maximum luminance decreases. As shown in Figure 2b, the blue curve represents the original horizontal viewing angle curve; the red curve represents the viewing angle curve with 85% of the maximum brightness as the threshold; and the green curve represents the viewing angle curve with 75% of the maximum brightness as the threshold. The angles *θ*_*−85%*_ and *θ_85%_* are smaller than *θ*_*−75%*_ and *θ_75%_* and larger than the original. Simultaneously, the maximum luminance of *θ_85%_* is larger than *θ_75%_* and smaller than the original. In the design example proposed in this paper, the maximum luminance reaches 85% of the original maximum luminance after widening the viewing angle.

**Figure 3:**
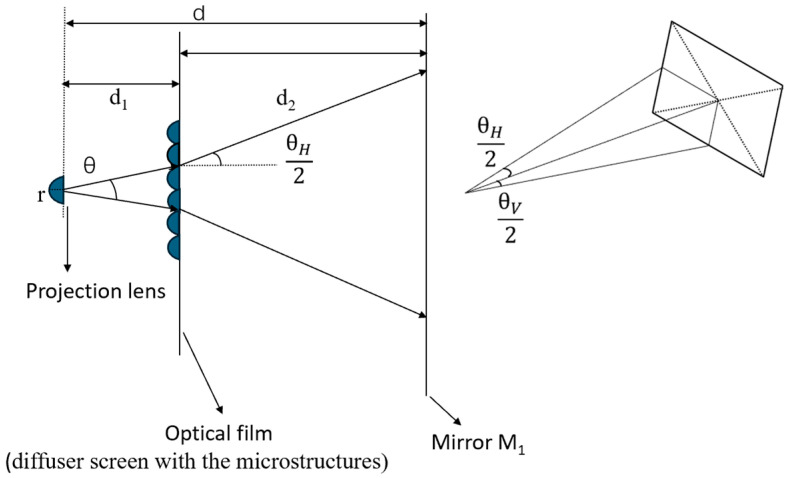
Schematic diagram of design parameters.

Figure 3 illustrates the design parameters of the microstructures, where *r* is the radius of the projection lens; *d*_1_ is the distance from the projection lens to the optical film; *d_2_* is the distance from the optical film to mirror M_1_; and *d* is the total distance from the projection lens to mirror M_1_, so *d* = *d*_1_ + *d*_2_. Set *θ* as the viewing angle for the rays emitted by the original projection lens and *θ_H_* and *θ_V_* as the required horizontal and vertical viewing angles, respectively, after the rays pass through the optical film.

**Figure 4:**
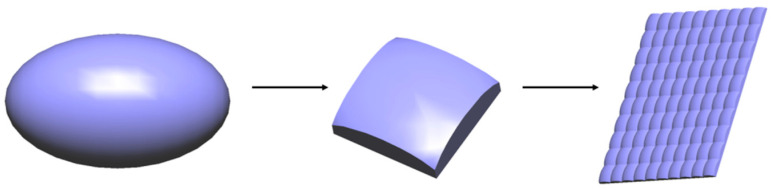
Schematic diagram of microstructure preparation process.

Considering that the major and minor axes of an ellipsoid on a single entity can have different curvatures, the original microstructure morphology is set as an ellipsoid, which allows for different control along the major and minor axes, thereby enabling independent regulation of the horizontal and vertical viewing angles. In addition, the ellipsoid must be cut into a rectangle with length *D_H_* and width *D_V_* to array the microstructures, as shown in Figure 4. The following section will explain in detail how to design the curvatures of the ellipsoid’s major and minor axes.

The rays emitted by the projection lens travel the distance of *d* to reach the mirror M₁, where they form an image with length *L_total_* and width *W_total_*. The image size *L_total_* and *W_total_* are determined by related angles, as well as the relative positions between projection lens M_1_ and the optical film. According to geometric relationship, there exists the following:

(1) $$\left\{\begin{array}{c} L_{total}=2\left(d_{1}tan\frac{\theta }{2}+d_{2}tan\frac{\theta_{H}}{2}\right)\\ W_{total}=2\left(d_{1}tan\frac{\theta }{2}+d_{2}tan\frac{\theta_{v}}{2}\right) \end{array}\right.$$

The image on the mirror M_1_ is regulated by the microstructure array with the resolution of *N_H_* × *N_V_* on the optical film. For each microstructure with the length of *D_H_* and the width of *D_V_*, *N_H_* and *N_V_* can therefore be calculated by Equation (2).

(2) $$\left\{\begin{array}{c} N_{\;H}=\frac{2{(d}_{1}tan\frac{\theta }{2}+r)}{D_{H}}\\ N_{V}=\frac{2{(d}_{1}tan\frac{\theta }{2}+r)}{D_{V}} \end{array}\right.$$

**Figure 5:**
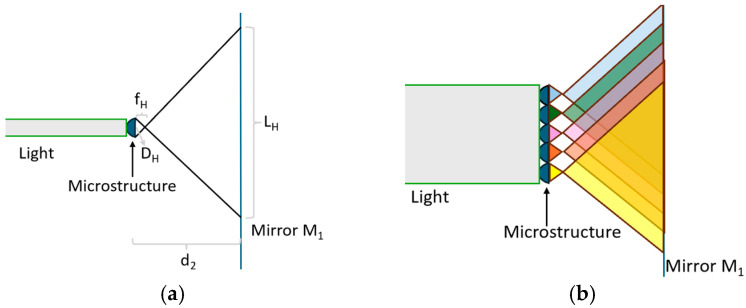
Schematic diagram of focal length calculation. (**a**) Overall analysis; (**b**) Single analysis.

When only considering a single microstructure, as shown in Figure 5a, the spot size on Mirror M_1_ is *L_single_* and *W _single_*. In addition, there exists Equation (3).

(3) $$\left\{\begin{array}{c} L_{\;single}=\frac{L_{total}}{D_{H}}\\ W_{single}=\frac{W_{total}}{D_{V}} \end{array}\right.$$

However, when there is a microstructure array on the optical film, the spot regulated by each microstructure could be overlapped, as shown in Figure 5b. *L_single_* and *W_single_* should be modified according to Equation (4) and denoted as *L’_single_* and *W’_single_*.

(4) $$\left\{\begin{array}{c} {L\prime }_{\;single}=L_{total}-(N_{H}-1)D_{H}\\ {W\prime }_{single}=W_{total}-(N_{V}-1)D_{V} \end{array}\right.$$

According to geometric relationship, there exists Equation (5).

(5) $$\left\{\begin{array}{c} \frac{D_{H}}{f_{H}}=\frac{{L\prime }_{single}}{d_{2}-f_{H}}\\ \frac{D_{V}}{f_{V}}=\frac{{W\prime }_{single}}{d_{2}-f_{V}} \end{array}\right.$$

where *f_H_* and *f_V_* represent the focal lengths along the major and minor axes, respectively.

The corresponding radius of curvatures *R_H_* and *R_V_* can therefore be derived, as shown in Equation (6).

(6) $$\left\{\begin{array}{c} R_{H}=f_{H}(n-1)\\ R_{V}=f_{V}(n-1) \end{array}\right.$$

where *n* is the refractive index of the microstructure material.

For an ellipsoid with the semi-major axis of *a*, the semi-minor axis of *b*, and the semi-height of *c*, there exists the following:

(7) $$\left\{\begin{array}{c} R_{H}=\frac{a^{2}}{c}\\ R_{V}=\frac{b^{2}}{c} \end{array}\right.$$

It is obvious that a series of configurations of *a*, *b*, and *c* can satisfy Equation (7). With the consideration that the ellipsoid should be large enough to make sure that it can be cut to create the rectangle with the size of *D_H_* and *D_V_*, *a* and *b* should satisfy Equation (8).

(8) $$\left\{\begin{array}{c} a\geq \frac{\sqrt{2}}{2}D_{H}\\ b\geq \frac{\sqrt{2}}{2}D_{V} \end{array}\right.$$

Based on the above design, the desired ellipsoid can be achieved. After cutting into rectangles, the microstructure array can be created, which will be prepared on the optical film.

## 3. Simulation Analysis and Discussion

To verify the proposed method, an example of the microstructure is designed. The regulated horizontal angle *θ_H_* and vertical angle *θ_V_* are set at 20° and 8°.

The parameters involved in the design are as follows: *θ* = 6°; *r* = 8 mm; *d_1_* = 60 mm; *d_2_* = 400 mm; *D_H_* and *D_V_* of a single microstructure = 0.03 mm; and *n* = 1.61.

According to Equation (1), the image dimensions at mirror M₁ can be calculated as length *L_total_* = 147.351 mm and width *W_total_* = 62.230 mm. The resolution of the microstructure array is 743 × 743. The dimensions of the image formed by a single microstructure at M₁ are as follows: length *L’_single_* = 125.092 mm and width *W’_single_* = 39.971 mm. Based on these dimensions, the focal length of each microstructure is calculated. The horizontal focal length *f_H_* and the vertical focal length *f_V_* are 0.096 mm and 0.300 mm, respectively. Subsequently, the corresponding radii of the curvature in the horizontal and vertical directions are derived as *R_H_* = 58.50 μm and *R_V_* = 18.30 μm, respectively. From this calculation, the following can be obtained: *a* = 39.34 μm, *b* = 22.00 μm, and *c* = 26.45 μm.

**Figure 6:**
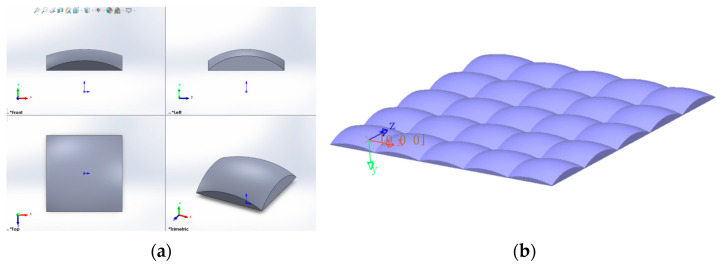
Profile of a single microstructure and microstructure array. (**a**) Single microstructure; (**b**) Microstructure array.

The profile of a single microstructure is shown in Figure 6, as well as a microstructure array.

**Figure 7:**
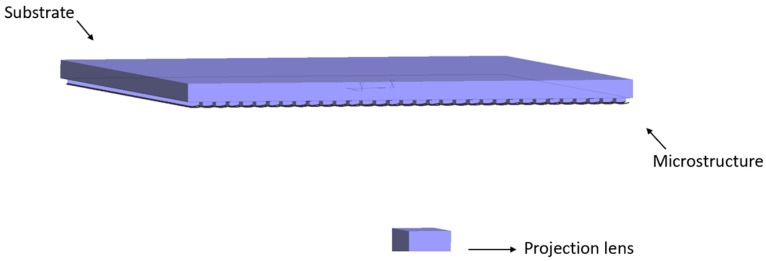
Simulation model.

Based on the calculated parameters, the simulation model was established in the optical simulation design software LightTools 8.4, as shown in Figure 7. The model includes an optical film consisting of a substrate and microstructures, a projection lens, and a receiving surface. The radius of the projection lens is set as *r* = 8 mm; the distance from the projection lens to the optical film is 60 mm; the distance from the optical film to the receiving surface is 400 mm; and the thickness of the optical film is 0.2 mm.

**Figure 8:**
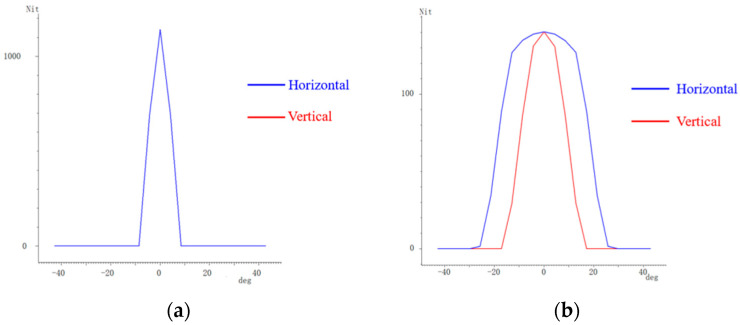
Viewing angle curves with and without optical film. (**a**) Without optical film; (**b**) With optical film.

The simulation results with and without the optical film are illustrated in Figure 8. Without the optical film, the horizontal and vertical viewing angle curves are completely identical, as shown in Figure 8a. The angle for both at 85% of the maximum luminance is 2°. With the optical film, the horizontal and vertical viewing angle curves becomes distinct. The angles at 85% of the maximum luminance on the horizontal and vertical viewing angle curves are 20° and 8°, respectively.

## 4. Preparation and Experimental Testing

### 4.1. Preparation of Optical Film

**Figure 9:**
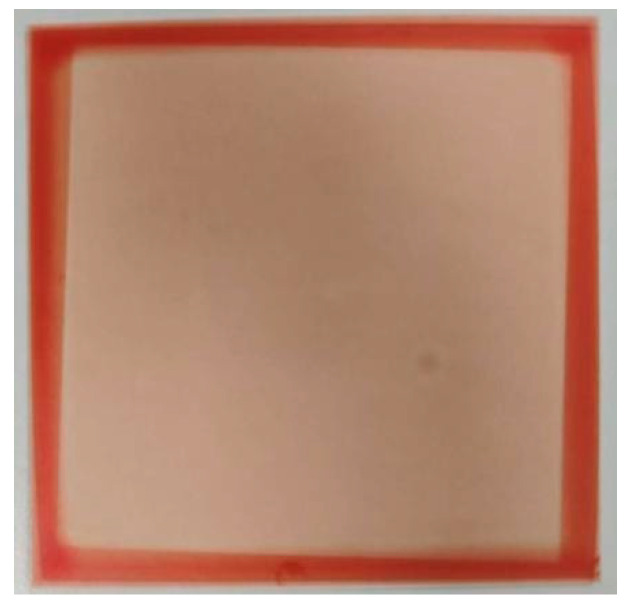
Physical diagram of optical film.

Based on the above design, the optical film with the designed microstructures was prepared using Heidelberg’s MLA100 maskless photolithography system (Heidelberg, Germany). First, the PET substrate was cleaned to remove surface dust and impurities. Subsequently, the photoresist was spin-coated onto the cleaned PET substrate. After that, the substrate with the spin-coated photoresist was dried to solidify the photoresist. Next, the photoresist was exposed according to the designed pattern. After exposure, the photoresist was developed to remove the excess photoresist, and the designed microstructure profile was obtained on the surface. Then, the substrate with the developed photoresist was rinsed and dried. Finally, a completed optical film with the designed microstructure was formed, as shown in Figure 9.

**Figure 10:**
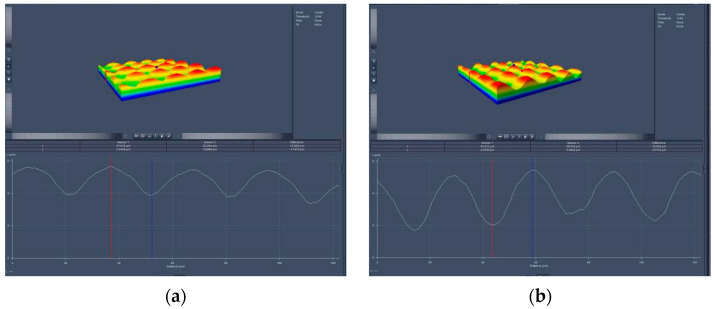
Practical microstructures observed with confocal microscope. (**a**) major axis direction; (**b**) minor axis direction. Colors vary according to different heights, displaying sequentially from blue to green, yellow, and red as the height increases from low to high.

The practical microstructures were observed using a confocal microscope (LSM700) from Carl Zeiss Company (Oberkochen, Germany), as shown in Figure 10.

### 4.2. Imaging Test

**Figure 11:**
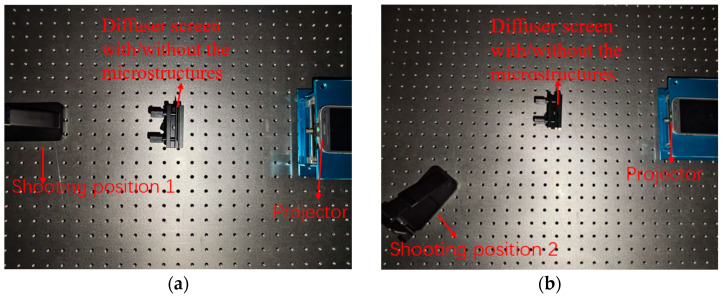
Experimental setup schematic. (**a**) major view; (**b**) oblique view.

To verify the influence of the optical film on imaging, we set up a simple experimental platform. As shown in Figure 11, it included a projector and a diffuser film with the microstructures. For comparison, a diffuser film without the microstructures was also tested.

**Figure 12:**
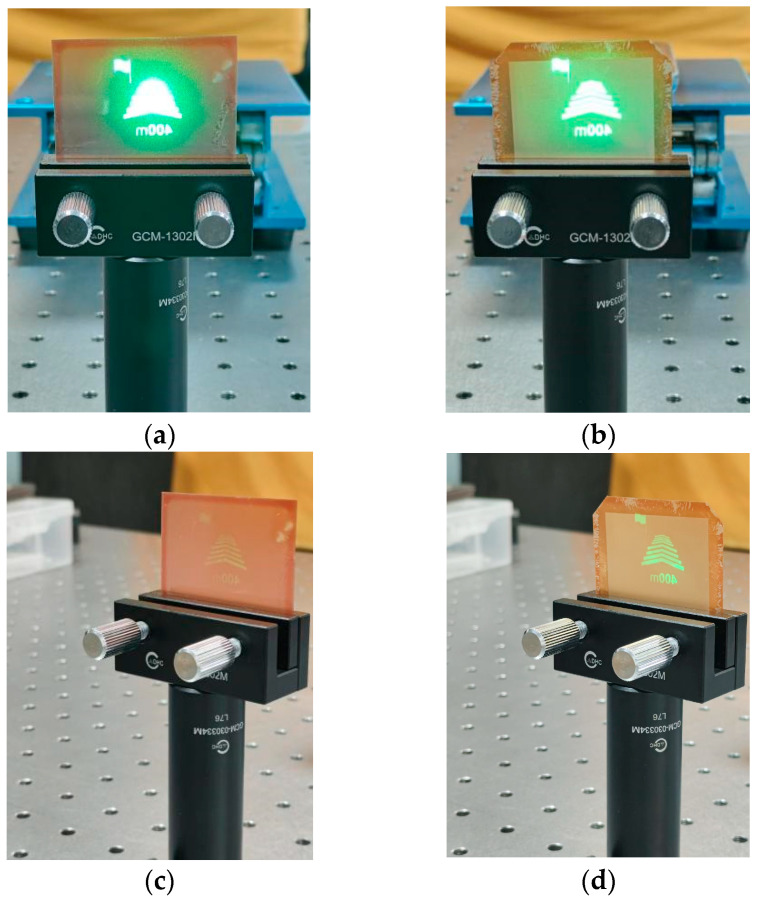
Images captured of (**a**) diffuser screen without microstructures at position 1; (**b**) diffuser screen with microstructures at position 1; (**c**) diffuser screen without microstructures at position 2; (**d**) diffuser screen with microstructures at position 2.

Figure 12 shows the image effects under varying conditions, with the projection lens’ luminous intensity and its distance to the diffuser screen fixed, while only the diffuser screen type and shooting position change. When comparing Figure 12a with Figure 12b, the image quality of the diffuser film with the microstructures shows no significant degradation compared to that without microstructures. The same conclusion can be drawn from comparing Figure 12c,d. Analysis of Figure 12c,d reveals that the brightness at wide viewing angles is significantly higher for the diffuser film with microstructures than for the one without.

The experimental results show that the image quality with microstructures is almost same as without microstructures. Simultaneously, the horizontal viewing angle is effectively widened.

### 4.3. Viewing Angle Test

**Figure 13:**
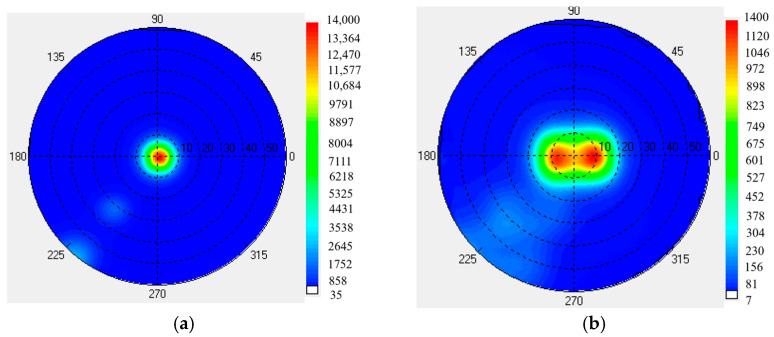
Viewing angle luminance map. (**a**) Without optical film; (**b**) With optical film.

The viewing angle luminance map was measured—using the EZ-LITE viewing angle tester from ELDIM Company (Caen in Normandy, France)—both with and without the optical film, as shown in Figure 13.

**Figure 14:**
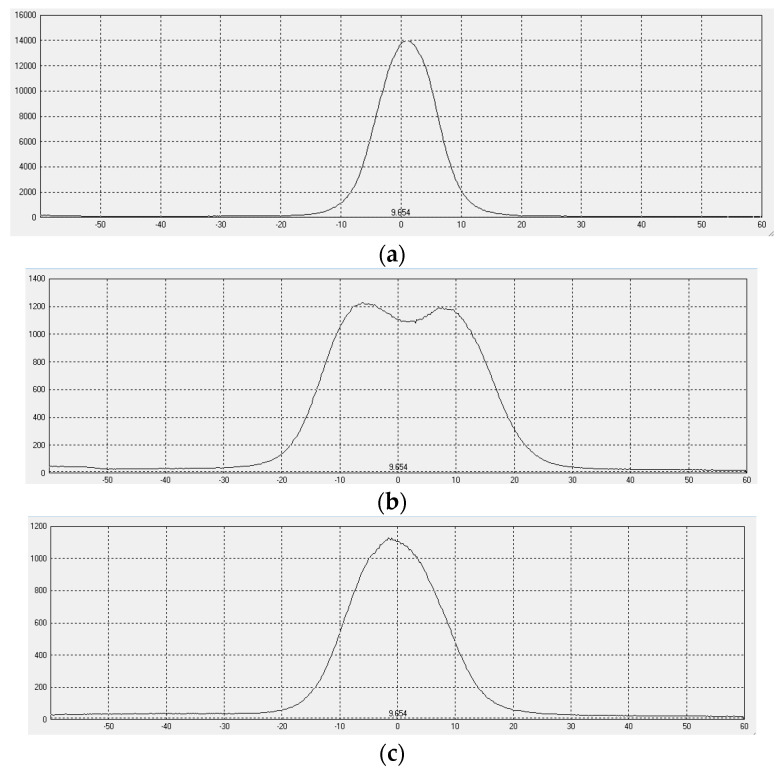
Viewing angle curves. (**a**) Without optical film; (**b**) Horizontal viewing angle curve with optical film; (**c**) Vertical viewing angle curve with optical film.

The horizontal and vertical viewing angle curves are shown in Figure 14. The tested horizontal and vertical viewing angles exhibit significant differences. The horizontal viewing angle corresponding to 85% of the maximum luminance increases from 2° without the film to 23° with the film. Meanwhile, the tested vertical viewing angle increases from 2° to 10°.

When compared with the simulated viewing angle of 20°, there is a 3° difference in the practical viewing angle. This discrepancy may result from differences in haze between the simulated and practical diffuser films. For the practical optical film, the microstructures are prepared on a diffuser film that is commonly used in backlight units for LCD. Precisely controlling the haze of the practical diffuser film is challenging, which may contribute to the difference between the simulated and experimental results.

## 5. Conclusions

This study presents a method for independently regulating the horizontal and vertical viewing angles. By utilizing the differing curvatures of an ellipsoid’s major and minor axes, the designed microstructure effectively expand the horizontal viewing angle while keeping the vertical viewing angle narrow, thereby aligning with practical driving demands for wider horizontal visibility. Both the simulated and experimental results confirm that the optical film enables the independent regulation of horizontal and vertical viewing angles—widening the horizontal viewing angle at 85% of maximum luminance from 2° to 20° (simulation) and to 23° (experiment), while maintaining the vertical viewing angle at 8° (simulation) and 10° (experiment). These results demonstrate the feasibility of independent viewing angle adjustment, addressing the limitations of conventional approaches.

## Data Availability

The data that support the findings of this study are available from the corresponding authors upon reasonable request.
